# Insights into the *In Vivo* Regulation of Glutamate Dehydrogenase from the Foot Muscle of an Estivating Land Snail

**DOI:** 10.1155/2012/317314

**Published:** 2012-03-26

**Authors:** Ryan A. V. Bell, Neal J. Dawson, Kenneth B. Storey

**Affiliations:** Department of Chemistry, Carleton University, 1125 Colonel By Drive, Ottawa, ON, Canada K1S 5B6

## Abstract

Land snails, *Otala lactea*, survive in seasonally hot and dry environments by entering a state of aerobic torpor called estivation. During estivation, snails must prevent excessive dehydration and reorganize metabolic fuel use so as to endure prolonged periods without food. Glutamate dehydrogenase (GDH) was hypothesized to play a key role during estivation as it shuttles amino acid carbon skeletons into the Krebs cycle for energy production and is very important to urea biosynthesis (a key molecule used for water retention). Analysis of purified foot muscle GDH from control and estivating conditions revealed that estivated GDH was approximately 3-fold more active in catalyzing glutamate deamination as compared to control. This kinetic difference appears to be regulated by reversible protein phosphorylation, as indicated by ProQ Diamond phosphoprotein staining and incubations that stimulate endogenous protein kinases and phosphatases. The increased activity of the high-phosphate form of GDH seen in the estivating land snail foot muscle correlates well with the increased use of amino acids for energy and increased synthesis of urea for water retention during prolonged estivation.

## 1. Introduction

Glutamate dehydrogenase (GDH; E.C. 1.4.1.3) is an important enzyme that contributes to a diverse set of metabolic processes. GDH catalyzes the following reversible reaction within the mitochondrial matrix:

(1) $$\begin{array}{cc} & \text{L-glutamate}+\text{NAD}{\left(\text{P}\right)}^{+}\\ & \;\;\;\;\;\;\to \alpha \text{-ketoglutarate}+{{\text{NH}}_{4}}^{+}+\text{NAD}\left(\text{P}\right)\text{H}. \end{array}$$

Through oxidative deamination, GDH gates the entry of numerous amino acid carbon skeletons into the Krebs cycle for increased energy production or gluconeogenic output. Furthermore, GDH-derived ammonium ions provide the primary source of nitrogen for the synthesis of urea via the urea cycle. In the reverse direction, GDH acts to synthesize L-glutamate for use in protein synthesis or, alternatively, transamination reactions. Given the importance of GDH in both carbohydrate and nitrogen metabolism, it was hypothesized to be a critical enzyme in animals that experience drastic alterations to cellular biochemistry in response to harsh environmental conditions.

Animals that live in seasonally hot and dry environments usually require some mechanism to survive periodic droughts and the scarcity of food that typically follows. One such mechanism is estivation, which is a state of aerobic torpor that is employed by a range of organisms including amphibians, reptiles, small mammals, and land snails [1]. Estivation entails major behavioral, physiological, and biochemical adaptations that allow for prolonged survival under harsh conditions. Particularly important for this study are the reprioritization of energy metabolism, differential regulation of many enzymes, and a strong reduction in overall metabolic rate [2]. Many of the adaptations seen during this type of aerobic torpor are tailored to retaining water or rationing metabolic fuel reserves, two major problems for estivators.

 One of the well-studied estivators is the pulmonate land snail, *Otala lactea*, which is known to estivate for many months at a time and have developed a variety of mechanisms for retaining water and conserving fuel reserves. Evaporative water loss can be particularly problematic in arid conditions and is lessened in these snails through the secretion of a hard mucous membrane over the shell aperture [3], and intermittent breathing patterns [4]. An additional crucial mechanism for maintaining a sufficient amount of body water is the accumulation of osmolytes. *Otala lactea *elevate the levels of selected cellular metabolites, in particular urea, to increase the osmolarity of their tissues and retard water loss due to the colligative action of high solute levels in body fluids. Land snails can accumulate urea at concentrations as high as 150–300 mM during prolonged estivation [5]. The reliance of land snails, as well as other estivating species, on the production of urea increases the importance of nitrogen metabolism during this period.

Long-term survival also depends on minimizing the rate of consumption of fixed body reserves of metabolic fuels. This is achieved by two intertwined actions: an overall strong suppression of metabolic rate (ATP turnover), and a reorganization of the priorities for ATP use to minimize ATP consumption by nonessential metabolic processes while sustaining ATP availability for essential functions. For preserving energy, these snails reduce movements, decrease heart rate, and decrease their overall metabolic rate by 70–90% [6]. Although metabolic rate is greatly depressed, metabolism must continue to provide energy for essential cellular processes. During estivation *Otala lactea* use carbohydrates as the primary source of energy, with amino acid oxidation becoming increasingly more important as carbohydrate stores run out and as dehydration stress demands a build-up of urea [5]. Furthermore, there is a low level lipid oxidation that occurs throughout the dormancy period to also aid in energy production.

The present study reveals that purified GDH from the foot muscle of *Otala lactea* is differentially phosphorylated between control and estivating conditions, and that this may be the regulatory mechanism responsible for altering GDH kinetics during dormancy.

## 2. Materials and Methods

### 2.1. Animals

*Otala lactea* were imported from Morocco and were purchased from local seafood retailer. Several hundred snails were placed in large plastic tubs lined with damp paper towels. The snails were maintained there for approximately one month and were fed every 2-3 days with shredded cabbage and carrots (sprinkled with crushed chalk). After this period, estivation was induced in some of the snails (~40% of the overall population chosen at random) by placing them in a dry plastic container without food. Active snails remained in the damp container with food and were used as the controls. After 10 days, both control (active) and estivated snails were sacrificed; foot muscle was dissected out, quickly frozen in liquid nitrogen and then stored at −80°C until use.

### 2.2. Sample Preparation and Purification of GDH

 Frozen foot muscle samples were homogenized 1 : 5 w : v in cold homogenization buffer containing 50 mM Tris-HCl, pH 8.0, 10 mM 2-mercaptoethanol, 2.5 mM EDTA, 2.5 mM EGTA, 25 mM *β*-glycerol phosphate (*β*GP), and 10% glycerol. A few crystals of phenylmethylsulphonyl fluoride (PMSF) were added at the time of homogenization to inhibit serine proteases. Homogenates were centrifuged for 30 minutes at 13,500 ×g and the supernatant was decanted and held on ice until use.

 GDH from *Otala lactea* foot muscle was purified through affinity chromatography, specifically, a GTP-agarose column. Following tissue extract preparation, ~2 mL of foot muscle extract was applied to a GTP-agarose column (2.5 × 1 cm) equilibrated in homogenization buffer. Once the extract entered the column, the column was washed with 10 mL of homogenization buffer to remove any unbound material. GDH was then eluted off the column with a linear salt gradient of 0-1 M KCl (made in homogenization buffer). 750 *μ*L fractions were collected and subsequently assayed under optimal conditions for the glutamate-oxidizing reaction for GDH. The five most active fractions collected off of the GTP-agarose column were pooled for use in GDH kinetic analyses. The purity of the final preparation was assessed by running an SDS gel and subsequently staining with silver stain [7].

### 2.3. GDH Assays

Optimal (*V*_max⁡_) assay conditions for the purified *Otala lactea* foot muscle GDH in the glutamate-oxidizing direction were 50 mM L-glutamate, 1.5 mM NAD^+^, 0.5 mM Mg-ADP, and 50 mM Tris-HCl buffer, pH 8.0 in a total volume of 200 *μ*L with 50 *μ*L of purified extract used per assay. In the glutamate-synthesizing reaction, the optimal concentrations of substrates were 1 mM *α*-ketoglutarate, 100 mM NH_4_Cl, 0.2 mM NADH, 0.5 mM Mg-ADP, 50 mM HEPES buffer, pH 7.2 with 20 *μ*L of purified foot muscle extract used in each assay. In all cases GDH activity was assayed with a Thermo Labsystems Multiskan spectrophotometer (A_340_). Enzyme activity was analyzed with a Microplate Analysis Program [8] and kinetic parameters were determined using the Kinetics v.3.5.1 program [9].

### 2.4. Effectors of GDH

 The effect of ADP, ATP, GTP, and citrate on the glutamate-synthesizing reaction of GDH was assessed by measuring GDH activity at suboptimal concentrations of all substrates. The suboptimal concentrations were 0.2 mM *α*-ketoglutarate, 0.15 mM NADH, and 40 mM NH_4_Cl. The effects of the aforementioned effector molecules on GDH activity were determined by varying the concentration of these molecules in the assay wells and ultimately calculating their *I*_50_ or *K*_*a*_. Both *K*_*a*_ and *I*_50_ values were calculated using the Kinetics v.3.5.1 program [9]. For the glutamate-oxidizing reaction, the effect of ADP on GDH activity was assessed; suboptimal substrate concentrations were 10 mM L-glutamate and 0.5 mM NAD^+^.

### 2.5. ProQ Diamond Phosphoprotein Staining of GDH

The overall phosphorylation state of control and 10 day estivated GDH from the foot muscle of the land snail was assessed by staining an SDS-gel with ProQ Diamond phosphoprotein stain. Purified GDH extracts were mixed 1 : 1 with SDS loading buffer (100 mM Tris buffer, pH 6.8, 4% w : v SDS, 20% v : v glycerol, 0.2% w : v bromophenol blue, 10% v : v 2-mercaptoethanol) and then boiled for 5 minutes, cooled on ice and 0.5 *μ*g of protein was loaded on a 10% SDS gel. The gel was run at 180 V for 45 min in running buffer containing 25 mM Tris-base, 250 mM glycine, and 0.1% w : v SDS. The gel was then washed twice in fixing solution (50% v : v methanol, 10% v : v acetic acid) and left rocking overnight in fixing solution at ~4°C. The following day, the gel was washed three times with ddH_2_O for 5–10 min each and then stained with ProQ Diamond Phosphoprotein stain (Invitrogen, Eugene, OR, USA) for 90 min. During staining, and thereafter, the gel container was covered with tin foil to prevent light from reaching the light-sensitive stain. Following staining, the gel was destained by washing twice with ProQ Diamond destaining solution (20% v : v acetonitrile, 50 mM sodium acetate, pH 4) for 30 min each time. The gel was then washed three times with ddH_2_O for 5–10 min each. Fluorescent bands on the gel were visualized using the ChemiGenius Bioimaging System (Syngene, Frederick, MD, USA) and intensities were quantified using the associated GeneTools software. Following quantification, the gel was stained for 30 minutes with Coomassie Brilliant Blue (25% w/v Coomassie Brilliant Blue R in 50% v/v, 7.5% v/v acetic acid) and destained with destaining solution (60% v/v methanol, 20% v/v acetic acid in ddH_2_O). GDH band intensities from ProQ Diamond chemiluminescence were normalized against the corresponding gel stained for total protein to normalize for any variations in sample loading.

### 2.6. *In Vitro * Incubations to Stimulate Phosphorylation or Dephosphorylation of GDH

Crude foot muscle extracts were homogenized in homogenization buffer using the same protocol as stated above, with the ratio of the mass of frozen tissue to homogenization buffer was altered to 3 : 5. It was later returned to the concentration used in the previous sample preparation after the samples were subjected to a 24 h incubation period at 4°C with reagents to stimulate the activities of endogenous protein kinases or protein phosphatases within the crude samples. All incubations included a basic incubation buffer (50 mM Tris-HCl, 10% v : v glycerol, 10 mM *β*-mercaptoethanol, pH 8.0) as well as the following additions:

- control incubations (denoted as STOP): 2.5 mM EDTA, 2.5 mM EGTA, and 25 mM *β*GP;
- open incubations: contained just the basic incubation buffer;
- stimulation of endogenous protein kinases (denoted total kinase) activities: 5 mM Mg·ATP, 30 mM *β*GP, 1 mM cAMP to stimulate protein kinase A (PKA), 1 mM cGMP to stimulate protein kinase G (PKG), 1.3 mM CaCl_2_ + 7 *μ*g/mL phorbol myristate acetate (PMA) to stimulate protein kinase C (PKC), 1 mM AMP to stimulate AMP-dependent protein kinase (AMPK), and 1 U of calmodulin + 1.3 mM CaCl_2_ to stimulate calcium-calmodulin kinase activity (CaMK);
- stimulation of endogenous protein phosphatases (denoted total PPase) activities: 5 mM MgCl_2_ and 5 mM CaCl_2_.

Following incubation, each of the above incubation solutions were spun through G50 Sephadex beads, preequilibrated in STOP buffer, at 2500 rpm to remove any low molecular weight molecules that were present during the incubation. *V*_max⁡_ (with ADP) was subsequently determined for GDH from the foot of the control and 10 day estivated land snails.

### 2.7. Data, Statistics, and Protein Determination

The data presented are expressed as mean ± SEM from independent determinations on separate enzyme preparations, with control and 10 day estivated values being compared using the Student's *t*-test. The soluble protein concentration was determined by the Coomassie blue dye-binding method using the BioRad prepared reagent with bovine serum albumin as the standard.

## 3. Results 

### 3.1. GDH Purification and Kinetics

**Figure 1:**
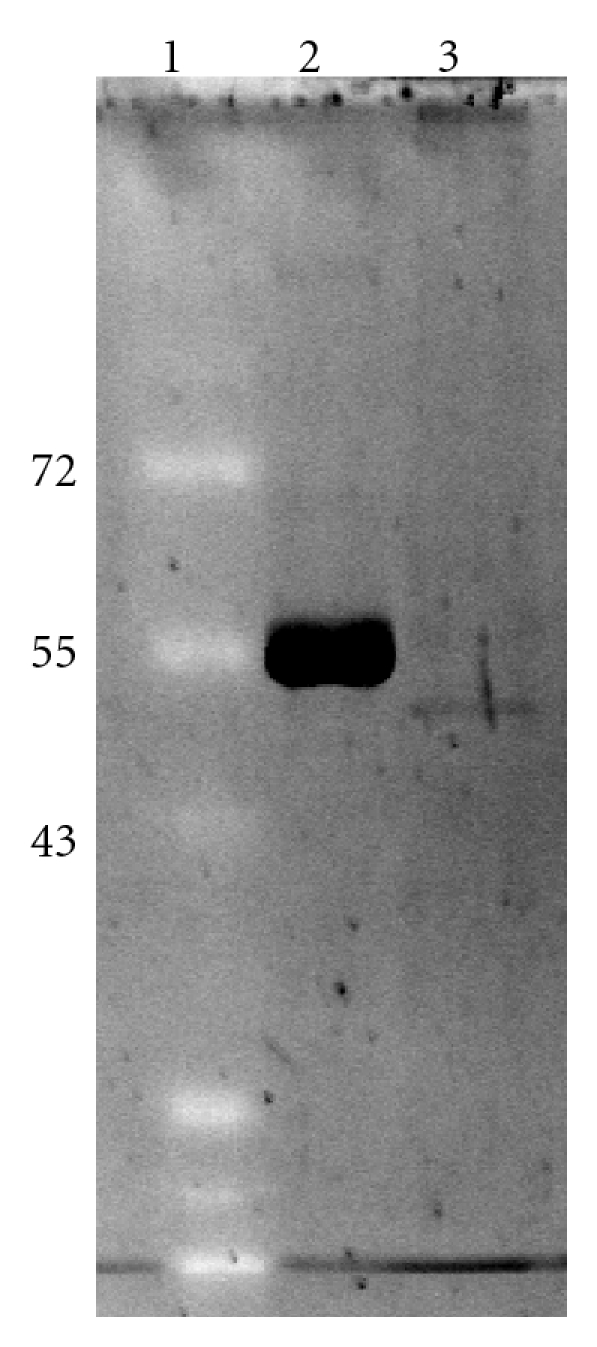
Purified control *Otala lactea* foot muscle GDH. The silver-stained gel displays the following: lane 1: Thermo Scientific Fermentas protein ladder with the molecular weight (kDa) of key bands indicated to the left of the lane; lane 2: bovine liver GDH (Sigma); lane 3: purified control land snail GDH.

 GDH was purified to electrophoretic homogeneity (Figure 1) using a GTP-agarose affinity column. This one step purification isolated GDH with a yield of 64% and a fold purification of 2000 (Table 1). GDH was stable for more than 48 hr (stored at 4°C) following purification without appreciable loss of activity (data not shown). 

Following purification, foot muscle GDH kinetics in the glutamate-oxidizing and glutamate-synthesizing directions were analyzed. In the glutamate-oxidizing reaction, several kinetic parameters were significantly different (*P* < 0.05) between active and estivated land snails. For instance, *K*_*m*_ glutamate (with ADP) decreased by 30% and the *V*_max⁡_ in the absence of ADP was >3-fold higher for estivated GDH as compared to the value for the control enzyme (Table 2). Similarly, in the presence of ADP the maximal velocity of estivated GDH was ~2-fold higher than the *V*_max⁡_ for control GDH. 

 The glutamate-synthesizing reaction of GDH also displayed distinctly different kinetics between control and 10 d estivated land snails.  Table 3 shows that *K*_*m*_ *α*-ketoglutarate and *K*_*m*_  NH_4_^+^ were ~3-fold and 2-fold higher, respectively, for estivated GDH as compared to GDH from active snails. Furthermore, the maximal velocity of GDH in the absence of ADP was 78% lower during estivation in comparison to the control *V*_max⁡_. The *V*_max⁡_ of GDH in the presence of ADP also decreased during estivation, with the maximal velocity being 56% lower than the corresponding control value. 

 Interestingly, the ratios of maximal velocities (glutamate-oxidizing reaction *V*_max⁡_/glutamate-synthesizing reaction *V*_max⁡_) were very different between control and 10 d estivated *Otala lactea*. Estivated GDH *V*_max⁡_ ratio in the absence of ADP was ~14-fold greater than the corresponding ratio for GDH from active control snails (Table 4). Similarly, in the presence of ADP, the *V*_max⁡_ ratio for GDH from estivating snails was ~5-fold greater than the control *V*_max⁡_ ratio. 

### 3.2. Effectors of GDH

 The effects of common intracellular molecules on the glutamate-oxidizing and glutamate-synthesizing reactions of GDH were determined by measuring enzyme activity at various concentrations of the effector molecules. The only molecule tested on the glutamate-oxidizing reaction was ADP and it behaved as an activator. Although the *K*_*a*_ ADP did not change between control and estivated GDH, the fold activation caused by the addition of ADP was 78% less for estivated GDH as compared to the control GDH fold activation (Table 2). 

 For the glutamate-synthesizing reaction, the effects of AMP, ADP, ATP, GTP, and citrate on foot muscle GDH activity were evaluated. Similar to the situation for the glutamate-oxidizing reaction, ADP was an activator of GDH. The *K*_*a*_ ADP and the corresponding fold activation of GDH were not significantly different between active and estivated conditions (Table 3).  Table 5 shows the actions of the other nucleotides. The addition of AMP to GDH assays activated the control enzyme form at low millimolar concentrations, but GDH from estivated snails was unaffected by AMP at the concentrations up to 4.5 mM. Similarly, the addition of ATP inhibited the control enzyme while having no effect on estivated GDH up to a concentration of 10 mM. On the other hand, GTP inhibited GDH from both the estivated and active conditions. GDH from the estivating snails displayed an *I*_50_ GTP that was 56% lower than the same value for active snails. Lastly, the addition of citrate at concentrations up to 10 mM had no effect on GDH from either control or estivating conditions. 

### 3.3. Reversible Phosphorylation of GDH

**Figure 2:**
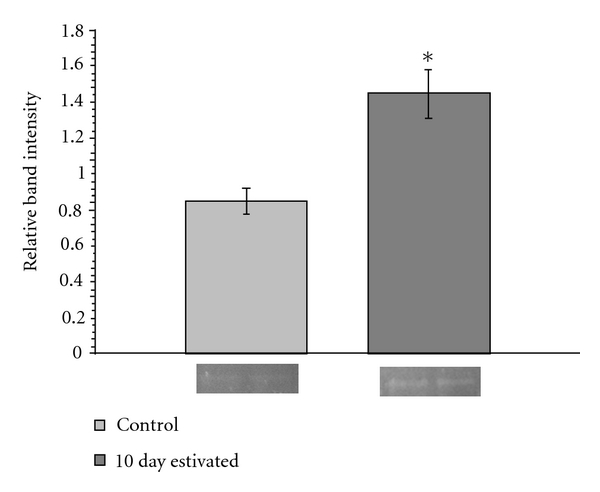
*Otala lactea *foot muscle GDH phosphorylation state. The relative band intensity of purified foot muscle GDH from control and 10 day estivated conditions after staining with ProQ Diamond phosphoprotein stain. Data are means ± SEM, *n* = 3 independent purified preparations. Example bands are shown below their corresponding bar. *Significantly different from the relative band intensity of the control condition using the Student's *t*-test (*P* < 0.05).

 To determine if the stable kinetic changes in GDH between active and estivating land snails were due to reversible phosphorylation, purified extracts were subjected to SDS-PAGE and subsequently stained with ProQ Diamond phosphoprotein stain. Having partly purified GDH using its strong affinity for GTP, most other proteins were removed prior to electrophoresis. Thus, after electrophoresis the single band found at the correct molecular weight range for GDH subunits was attributed to GDH. Furthermore, when purified commercial bovine liver GDH (Sigma) was run on the same SDS-PAGE gels, it gave a band near the same position as the snail GDH subunits after ProQ Diamond staining.  Figure 2 shows that the quantified band intensity for estivated GDH was ~75% more intense (*P* < 0.05) than that observed for control GDH. 

**Figure 3:**
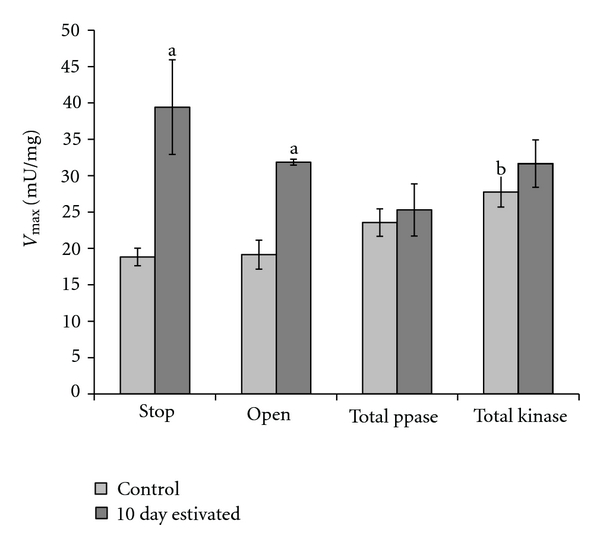
Effect of *in vitro* incubations to stimulate the activity of endogenous protein kinases and protein phosphatases on *Otala lactea *foot muscle GDH *V*_max⁡_. The STOP condition indicates where both protein kinases and phosphatases were inhibited during the incubation, and OPEN indicates an incubation that did not activate nor inhibit any protein kinases or phosphatases. Data are means ± SEM, *n* = 3 independent purified preparations. ^a^Significantly different from the corresponding control value under the same incubation condition. ^b^Significantly different from the corresponding value in the STOP condition. The statistical test used was the Student's *t*-test (*P* < 0.05).

To investigate the effect of an altered phosphorylation state on GDH kinetics, crude foot muscle extracts were incubated with solutions that would activate endogenous protein kinases or phosphatases and the maximal activity of the enzyme was subsequently determined. Control incubations, denoted STOP, maintained a similar difference in *V*_max⁡_ between control and 10 day estivated GDH prior to any incubation (Table 2 for preincubation *V*_max⁡_ and Figure 3 for *V*_max⁡_ after incubation). Incubations, denoted OPEN, which contained no activators or inhibitors of protein kinases or phosphatases showed a similar activity profile for control and 10 day estivated GDH as seen in the STOP condition. Stimulation of endogenous protein phosphatases through incubation with divalent cations led to a ~35% reduction in estivated GDH *V*_max⁡_ as compared to the same value from the STOP condition, however this was not shown to be statistically significantly different. The control form of the enzyme appeared unaffected by this same treatment. Conversely, after overnight stimulation of endogenous protein kinases, control GDH *V*_max⁡_ increased by ~35% as compared to control *V*_max⁡_ from the STOP condition. Estivated GDH *V*_max⁡_ appeared to be unaffected by this incubation, and maintained a value similar to that seen in the STOP condition. 

## 4. Discussion 

 The land snail, *Otala lactea*, typically inhabits arid lands where the environment can become very dry and food can become scarce. When confronted with these conditions, these snails enter into a state of aerobic torpor called estivation. This state is characterized by major biochemical changes which include the reorganization of fuel utilization, and increases in the concentration of osmolytes for water retention [2]. An enzyme that may contribute to these processes is glutamate dehydrogenase, which can produce *α*-ketoglutarate for aerobic energy production and NH_4_^+^ for ureogenesis. This investigation shows that foot muscle GDH from control and estivating land snails displays markedly different kinetic properties, responses to metabolites, and is regulated by reversible phosphorylation. 

 The initial kinetic studies of purified GDH revealed distinctly different stories for the glutamate-oxidizing and the glutamate-synthesizing reactions. In the glutamate oxidizing direction, GDH from the estivating land snail was more active than GDH from the control snails. Estivated GDH displayed a significantly lower *K*_*m*_ glutamate (with ADP), as well as significant increases in *V*_max⁡_ both with and without ADP as compared to control (Table 2). Conversely, estivated GDH in the reverse direction was significantly less active when compared to control. Evidence for this stems from the significant increases in *K*_*m*_ *α*-ketoglutarate, *K*_*m*_NH_4_^+^, and dramatic decreases in *V*_max⁡_ both with and without ADP for estivated GDH as compared to control GDH (Table 3). The degree to which the activity of the forward and reverse reactions change between control and estivated GDH is illustrated by the *V*_max⁡_ ratios represented in Table 4. These ratios show that under control conditions, GDH is more active in the glutamate-synthesizing reaction, while during dormancy GDH was substantially more active in the glutamate-oxidizing direction. 

The activation of the glutamate-oxidizing reaction of GDH observed in this study during estivation coincides with the biochemical changes that transpire in this hypometabolic state. One of the primary means for water retention during estivation in the land snail is the accumulation of metabolites to increase tissue osmolarity. Typically the increases in osmolarity are mediated by the production of urea (concentration can reach 150–300 mM in some land snail species) [5], which is derived mainly from the ammonium released by the oxidative deamination of glutamate by GDH. Increased glutamate breakdown via GDH and subsequent urea production during osmotic stress is not uncommon, and is present in a variety of species that encounter high-salinity or dry environments. For instance, studies with euryhaline anurans have shown that hypersalinity-induced urea biosynthesis was associated with increased GDH activity, in conjunction with increased activity of the urea cycle enzymes [10]. Furthermore, numerous species of Amphibia elevate levels of urea cycle enzymes and GDH to raise urea synthesis in moderately saline environments [11]. 

Urea production by the urea cycle is typically confined to the liver or liver-like organ of most animals, however there have been recent studies that indicate that extrahepatic urea production is possible. For instance, several fish species including the little skate (*Raja erinacea*) [12], the spiny dogfish (*Squalus acanthias*) [13], the Lake Magadi tilapia (*Alcolapia grahami*) [14], and the largemouth bass (*Micropterus dolomieu*) [15] display significant amounts of urea cycle enzyme activity in muscle and/or intestinal tissues. Interestingly, some of these animals have shown increased extrahepatic urea synthesis during periods of stress. For instance, Saha et al. [16] proposed that extrahepatic urea production may be important in the walking catfish (*Clarias batrachus*) during habitat drying, similar to that seen during snail estivation. Although the activity of the urea cycle enzymes and the urea content of *Otala lactea* foot muscle during estivation are unknown, muscle urea content is known to increase in the estivating Giant African snail, *Achatina fulica* [17]. Thus, it is plausible that activation of the ammonia-producing GDH reaction in the snail foot muscle might contribute to the accumulation of urea for the purpose of water retention during estivation. Further studies on the presence and activity of urea cycle enzymes in *Otala lactea* foot muscle would have to be conducted to reveal if this actually occurs *in vivo*. 

Another possibility for muscle-borne ammonia is that it could be exported into the hemolymph and transported to another organ for processing into urea. Muscle tissue is generally known to both release and take in ammonia from the blood in vertebrates [18], and this likely extends to invertebrates since ammonia transporters are well known in both prokaryotes and eukaryotes [19]. Once transported into the hemolymph the ammonia may be taken up by the hepatopancreas (as occurs in vertebrate liver) and subsequently processed into urea. 

 Although the buildup of urea during estivation is advantageous to prevent water loss, it may negatively affect enzyme function due to its ability to disrupt enzyme secondary structure [20]. Typically those organisms that accumulate urea combat its potentially harmful effects by (1) developing urea-adapted proteins and/or (2) counteracting the effects of urea with other metabolites. In the latter case, the counteracting metabolites are typically methylamines that stabilize macromolecules when present at sufficient levels in the cell [21]. Direct measurement of methylamines have not been determined for *Otala lactea*, however, studies on the estivating Mountain snail indicate that only low levels of counteracting metabolites are present during estivation [5]. Thus, it is likely that the land snails have evolved enzymes that can withstand high concentrations of urea in the absence of any counteracting agents. 

 In addition to the possible role of foot muscle GDH in urea production, it is likely that the activation of the glutamate-oxidizing reaction contributes to aerobic energy production during estivation. Fuel metabolism during land snail estivation changes over time, carbohydrates being oxidized primarily with amino acid catabolism becoming increasingly important as desiccation stress rises and carbohydrate stores are depleted [5]. The tissues used in this study were taken from snails that had been estivating for 10 days. At this early stage in estivation it is likely that the majority of the energy produced in cells was still derived from carbohydrate oxidation with small amounts of energy derived from protein and lipid catabolism. Thus, further activation of GDH may occur in the later stages of estivation, where the snail would rely more heavily on glutamate oxidation for energy production. 

 The reduced activity of the glutamate-synthesizing reaction during land snail estivation also coincides with the typical biochemical changes observed in this hypometabolic state. For instance, estivation involves a large reduction in metabolic rate, and this typically requires a suppression of most anabolic processes. Particularly important is the suppression of protein synthesis, which if maintained at normal levels during estivation would be impossibly costly [1]. Indeed, studies on numerous estivating species, including some frogs and pulmonate land snails, show a drastic decrease in protein synthesis [22]. Thus, it makes sense that the synthesis of glutamate via GDH, presumably for the eventual synthesis of proteins, is suppressed during land snail estivation. It is interesting to note, however, that glutamate levels have been known to rise in some species (i.e., the spadefoot toad) during estivation. However, this increase in glutamate concentration, as well as the concentration of some other amino acids, during estivation is typically associated with protein breakdown rather than amino acid synthesis [23]. 

 The kinetic differences outlined above indicate that GDH may be regulated when the animal transitions from its active condition to the state of aerobic torpor. One of the most common mechanisms for enzyme regulation is posttranslational reversible phosphorylation of an enzyme. To investigate the overall phosphorylation state of GDH from the control and 10 day estivated foot muscles, purified GDH was run on SDS-PAGE and stained by ProQ Diamond phosphoprotein stain. Figure 3 indicates that GDH from the estivating animal is significantly more phosphorylated than the control enzyme. Subsequent incubations that stimulated endogenous protein kinases and protein phosphatases within the crude extracts, revealed that activating protein kinases increased control GDH *V*_max⁡_, but had little effect on estivated GDH *V*_max⁡_. Conversely, stimulation of endogenous protein phosphatases did not significantly change either control or estivated GDH *V*_max⁡_, although estivated GDH *V*_max⁡_ was ~35% lower than the corresponding value for the STOP condition. Thus, it appears that modifying GDH phosphorylation state alters the activity of the enzyme, and may be the mechanism responsible for the kinetic changes observed in this study when the animal enters aerobic torpor. 

 Bioinformatic analysis of GDH from another gastropod mollusk, *Haliotis discus* (the sea snail) reveals that GDH might be capable of being phosphorylated by a number of kinases, such as calcium/calmodulin kinase 2, protein kinase C, Akt, and possibly casein kinase 2 (http://scansite.mit.edu/). Indeed, GDH from several organisms, including yeast [24, 25], *Escherichia coli* [26], and most recently in Richardson's ground squirrels [27] has been found to be phosphorylated. In the latter example, GDH activity in the glutamate-deaminating direction increased significantly during hibernation, with GDH found in the hibernating ground squirrel liver being less phosphorylated as compared to the euthermic control. This is contrary to that effect of phosphorylation found in this study, thus indicating that GDH is likely regulated in a stress- and/or tissue-specific manner. Indeed, different environmental insults are well known to cause varied changes in phosphorylation state of a particular enzyme as different protein kinases and phosphatases are activated in response to different environmental changes. For instance, creatine kinase in hibernating ground squirrel (*Spermophilus richardsonii*) skeletal muscle showed an increased level of phosphorylation that was accompanied by a decreased affinity for creatine [28], whereas increased phosphorylation of wood frog (*Rana sylvatica*) skeletal muscle creatine kinase was associated with an increased affinity for creatine [29]. Thus, while GDH regulation by phosphorylation may be essential for many animals that enter into a hypometabolic state, its regulation likely varies depending on the environmental stress encountered by the organism. 

 Regulation of metabolic enzymes through changes in phosphorylation state during *Otala lactea* estivation has been reported previously. Several glycolytic enzymes have been found to be regulated in this fashion in the land snail, including glycogen phosphorylase [30], phosphofructokinase [31], pyruvate kinase [32], and pyruvate dehydrogenase [33]. Furthermore, reversible phosphorylation has also been identified as the regulatory mechanism for enzymes outside glycolysis including glucose-6-phosphate dehydrogenase and the Na^+^/K^+^-ATPase membrane ion pump [34, 35]. Many of these enzymes exist in a high-phosphate form during estivation, which correlates well with the phosphorylation of foot muscle GDH during land snail estivation found in this study. 

The existence of several metabolic enzymes in a high-phosphate form during estivation suggests that protein kinases must be active during the early stages of estivation, and possibly throughout the prolonged dormant period. Although the individual protein kinases that act on GDH *in vivo* have not been investigated in this study, previous studies on protein kinases in *Otala lactea* indicate which kinases may phosphorylate GDH during estivation. For instance, Brooks and Storey [36] determined that the levels of cyclic GMP rise during estivation in *Otala lactea*. An increased cGMP level subsequently activates protein kinase G (PKG) making it a possible regulator of GDH activity. Conversely, protein kinase A appears to decrease in activity during estivation and is unlikely to be part of GDH regulation *in vivo* [37]. 

 Similar to the situation with the protein kinases, very little work has been done on the role of protein phosphatases during estivation in *Otala lactea*. Work on estivation in the spadefoot toad could suggest the role of protein phosphatases during this hypometabolic state, however, the protein phosphatases displayed varied responses to estivation, which depended on the type of phosphatase and also the tissue in which it was located [1]. Unfortunately, few phosphatases were investigated in the skeletal muscle of the toad and thus would have a limited applicability to the phosphatases in the foot muscle of *Otala lactea*. 

 In addition to the effect of phosphorylation on GDH substrate kinetics, phosphorylation of GDH may have also altered the enzyme's responses to common cellular intermediates. For instance, both control and estivating GDH showed similar affinities for ADP in the glutamate-oxidizing reaction, however, the fold activation by ADP was significantly higher for the low-phosphate control form of GDH (Table 2). This difference may reflect the energy requirements in the two states; in the control state, cellular energy needs are high and a decrease in the energy state of the cell could necessitate a much greater GDH response to enhance glutamate oxidation as a fuel, mediated by ADP as a signal of low energy. Alternatively, during estivation where energy needs are low, a dramatic increase in GDH activity would likely cause a backup of the TCA cycle as oxygen consumption, and therefore ETC activity, is reduced during estivation [38]. This large increase in fold activation for the control form of GDH was not seen for the reverse reaction of this enzyme. Furthermore, the *K*_*a*_ ADP measured in the reverse reaction was not significantly different between active and estivating snails (Table 3). Muscle ADP concentrations are typically above the *K*_*a*_ values for both control and estivated GDH observed here, and thus are likely a factor in regulating GDH activity *in vivo*. 

In addition to ADP, the effect of other cellular energy molecules was assessed on the glutamate-synthesizing reaction of GDH (Table 5). For instance, estivated GDH was relatively unresponsive to the addition of AMP to the assays, whereas control GDH was activated at low millimolar concentrations. The AMP concentration in *Otala lactea* foot muscle is known to be 0.34 *μ*mol/g wet weight under control conditions (~0.34 mM), and 0.23 *μ*mol/g wet weight during dormancy (~0.23 mM) [39]. Based on these results, the intracellular foot muscle AMP concentrations would be high enough to activate the control enzyme but not the estivated enzyme in this study. In response to ATP, control GDH displayed an *I*_50_ ATP of 7 mM whereas estivated GDH was uninhibited at concentrations up to 10 mM. The ATP concentrations reported by Churchill and Storey [39] indicate that land snail muscle ATP concentrations are approximately 0.83 *μ*mol/g wet weight (~0.83 mM) under control conditions, and 1.16 *μ*mol/g wet weight (~1.16 mM) during estivation. With the control *I*_50_ ATP being so high it is likely that both of these enzymes are unaffected by ATP *in vivo*. Alternatively, GDH was highly responsive to GTP, and both control and estivated GDH were inhibited at low micromolar concentrations. It is important to note that estivated GDH displayed a significantly lower *I*_50_ GTP in comparison to the control enzyme. Although the *in vivo* concentration of GTP is unknown, sensitive measurements of guanylate levels in *Helix aspersa maxima* muscle showed undetectable amounts of GTP [40]. Thus, the importance of GTP for regulating GDH *in vivo* is unknown. That being said, the unresponsiveness of the glutamate-synthesizing reaction of estivated GDH to AMP and the significantly higher sensitivity to GTP inhibition coincides with a shutdown of the glutamate-synthesizing reaction during estivation. 

Another metabolite tested on the glutamate-synthesizing reaction of GDH was citrate, a common product of lipid oxidation. Since, a constant and low level of lipid oxidation occurs in *Otala lactea* during estivation, citrate accumulates to appreciable amounts. Citrate is known to inhibit some metabolic enzymes, such as phosphofructokinase and glucose-6-phosphate dehydrogenase in the land snail during estivation and anoxia [31, 34]. Citrate is thought to bind to allosteric sites on PFK which have a similar structure to allosteric ADP binding sites [41]. Knowing the effect of citrate on some metabolic enzymes found in the land snail and its possible binding to ADP-binding sites, the effects of this metabolite were investigated on GDH activity in this study.  Table 5 shows that neither control nor estivated GDH showed any significant response to the addition of citrate up to 10 mM, and therefore this metabolite does not appear to be a significant regulator of GDH activity *in vivo*. 

 The kinetic changes elucidated in this study strongly suggest that foot muscle GDH exists in two different forms under control and estivating conditions in the land snail, *Otala lactea*. As revealed by ProQ Diamond phosphoprotein staining and incubations that stimulated protein phosphatases and kinases, these kinetic changes appear to be due to reversible phosphorylation of GDH. The differently phosphorylated forms display distinctly different behaviors, with the glutamate-oxidizing reaction being activated and the glutamate-synthesizing reaction inhibited for estivated GDH in comparison to control GDH. This correlates well with the increased use of amino acids for energy, as well as the increased synthesis of urea for water retention during prolonged estivation. 

## Figures and Tables

**Table 1:** Control *O. lactea* foot muscle GDH purification.

Purification step	Total protein (mg)	Total activity (mU)	Specific activity (mU/mg)	Fold purification	% yield
Crude extract	31	5.6	0.18	—	—
GTP-agarose	0.01	3.6	360	2000	64

**Table 2:** Comparison of purified foot muscle GDH kinetic parameters from control and 10 day estivated *Otala lactea* assayed in the glutamate-utilizing direction. Data are means ± SEM, *n* ≥ 3 independent purified preparations. *K*_*m*_ values were determined at optimal (i.e., those used to obtain *V*_max⁡_) cosubstrate concentrations. *K*_*a*_ values were determined at suboptimal substrate concentrations (identified in Section 2). ^a^Significantly different from the corresponding control value using the Student's *t*-test, *P* < 0.05. ^b^Significantly different from the same condition without ADP, *P* < 0.05.

	Control	10-day estivated
*K*_*m*_ glutamate (mM)	0.90 ± 0.02	0.84 ± 0.02
*K*_*m*_ glutamate with 0.5 mM ADP (mM)	3.0 ± 0.2^b^	2.1 ± 0.2^ab^
*K*_*m*_ NAD^+^ (mM)	0.59 ± 0.08	0.49 ± 0.02
*K*_*m*_ NAD^+^ with 0.5 mM ADP (mM)	0.159 ± 0.005^b^	0.191 ± 0.009^b^
*K*_*a*_ ADP (*μ*M)	4.7 ± 0.7	3.5 ± 0.5
Fold activation with ADP	13.8 ± 0.8	3.0 ± 0.4^a^
*V*_max⁡_ (mU/mg)	9.4 ± 0.8	30 ± 1^a^
*V*_max⁡_ with 0.5 mM ADP (mU/mg)	26.9 ± 0.2^b^	58 ± 3^ab^

**Table 3:** Comparison of purified foot muscle GDH kinetic parameters from control and 10 day estivated *Otala lactea* assayed in the glutamate-synthesizing direction. Data are means ± SEM, *n* ≥ 3 independent purified preparations. *K*_*m*_ values were determined at optimal (i.e., those used to obtain *V*_max⁡_) cosubstrate concentrations. *K*_*a*_ values were determined at suboptimal substrate concentrations (defined in Section 2). ^a^Significantly different from the corresponding control value using the Student's *t*-test, *P* < 0.05. ^b^Significantly different from the same condition without ADP, *P* < 0.05.

	Control	10-day estivated
*K*_*m*_ *α*-ketoglutarate (mM)	0.19 ± 0.03	0.55 ± 0.05^a^
*K*_*m*_ *α*-ketoglutarate with 0.5 mM ADP (mM)	0.22 ± 0.01	0.27 ± 0.04^b^
*K*_*m*_ NH_4_^+^ (mM)	63 ± 2	126 ± 3^a^
*K*_*m*_ NH_4_^+^ with 0.5 mM ADP (mM)	36 ± 1^b^	30 ± 3^b^
*K*_*a*_ ADP (*μ*M)	36 ± 4	27 ± 4
Fold activation with ADP	1.83 ± 0.07	1.63 ± 0.05
*V*_max⁡_ (mU/mg)	67 ± 4	15 ± 2^a^
*V*_max⁡_ with 0.5 mM ADP (mU/mg)	131 ± 4^b^	57 ± 3^ab^

**Table 4:** The ratios of maximal activity in the glutamate-oxidizing reaction to the maximal activity in the glutamate-synthesizing reaction for GDH from the foot muscle of active and 10-day estivated *Otala lactea*.

Control		10-day estivated	
*V*_max⁡_ ratio	*V*_max⁡_ ratio	*V*_max⁡_ ratio	*V*_max⁡_ ratio
(without ADP)	(with ADP)	(without ADP)	(with ADP)
0.14 ± 0.01	0.205 ± 0.006	2.0 ± 0.3	1.01 ± 0.07

**Table 5:** The effect of various metabolites on purified foot muscle GDH activity from control and 10 day estivated *Otala lactea*. GDH was assayed in the glutamate-synthesizing direction. *K*_*a*_ and *I*_50_ values were determined at suboptimal substrate concentrations (defined in the Section 2). Data are means ± SEM, *n* ≥ 3 independent purified preparations. *Significantly different from the corresponding control value using the Student's *t*-test, *P* < 0.05.

	Control	10-day estivated
*K*_*a*_ AMP (mM)	0.27 ± 0.04	No Effect (up to 4.5 mM)
*I*_50_ ATP (mM)	7 ± 1	No Effect (up to 10 mM)
*I*_50_ GTP (*μ*M)	0.34 ± 0.02	0.15 ± 0.02*
Citrate	No Effect	No Effect
